# Evaluation of Acute and Subacute Oral Toxicity Induced by Ethanolic Extract of *Marsdenia tenacissima* Leaves in Experimental Rats

**DOI:** 10.3390/scipharm85030029

**Published:** 2017-08-21

**Authors:** Mayur Porwal, Najam Ali Khan, Kamal Kishore Maheshwari

**Affiliations:** 1 Department of Pharmacology, Faculty of Pharmacy, IFTM University, Moradabad 244102, India; alikhan_najam@yahoo.co.in; 2 Department of Pharmacy, M.J.P. Rohilkhand University, Bareilly 243006, India; kamalbareilly@yahoo.co.in

**Keywords:** acute toxicity, biochemical analysis, hematological parameters, *Marsdenia tenacissima*, subacute toxicity, histopathology

## Abstract

The objective of this study is to evaluate the acute and subacute toxicity of the ethanolic extract of *Marsdenia tenacissima* (MTE) leaves (family: Asclepiadaceae) in albino rats. The acute toxicity was performed where the limit dose of 5000 mg/kg body weight used. Observations were made and recorded for 24 h, and once daily further for a period of 14 days. The rats were weighed and various observations, like mortality, behavior, injury, or any signs of illness were conducted once daily during the period. For subacute study, four groups of 10 animals (female rats) received 10% Tween 20 in distilled water (control), and 250, 500, and 1000 mg/kg of freshly-prepared extracts, respectively, every 24 h orally for 28 days. At the end of each study, hematological analysis and biochemical parameters were evaluated. Histopathological examination of vital organs of the animals were taken for gross findings, compared to controls. There was no significant difference (*p* > 0.05) observed in the relative organs, body weights, hematological, biochemical parameters, and gross abnormalities, compared to the control. No mortality was recorded. Therefore, analysis of results may lead to the conclusion that the medium-term oral administration of the MTE leaves for 28 days does not cause toxicity.

## 1. Introduction

Plant-derived medicines are used in all civilizations and cultures and, hence, plants have always played a key role in health care systems worldwide. In most developing countries, the indigenous modes of herbal treatment are a part of the culture and the dominant method of healing therapy. These remedies, with a considerable extent of effectiveness, are socially accepted, economically viable and, mostly, are the only available source [1]. Plants used in traditional medicine, therefore, have a critical role in the maintenance of health all over the world. The drugs of herbal, herbo-mineral, and animal origin have been used by the traditional healers to maintain health and treat diseases since antiquity. Such medicines are widely used in Africa and Asia, including India and China. Due to the adverse side-effects, and also the development of resistance against synthetic drugs, the uses of plant-derived drugs are becoming popular in developed countries also [2]. However, the latest surveys have indicated many medicinal plants also showed adverse effects [3]. This raises concerns about the potential toxic effect resulting from chronic use of such medicinal plants. Therefore, evaluating the toxicological effects of any medicinal plant extract intended to be used clinically or preclinically, is a crucial part of its assessment of potential toxic effects. 

*Marsdenia tenacissima* (MT) (Roxb.) Moon (English name: Rajmahal hemp; Hindi name: Marua bel; family: Asclepidaceae), is a large twining shrub found in the Himalayas from Kumaon to Assam up to an altitude of 1500 m, Indo-China, and West-China. It is an important medicinal plant having various pharmacological uses. MT have several pharmacological actions to a number of disorders, such as scurvy, urinary diseases, arthritis, heart disease, skin disease, thirst, pruritus, vomiting, and intermittent fever [4,5]. Different parts of the plants are used for various ailments. The stem possesses various pharmacological properties, like analgesic, anti-inflammatory, anti-arthritic [6], and antioxidant activities [7], while the roots have potent cytotoxic [8] and anti-HIV activity [9]. The traditional uses of MT are in abdominal pain due to worms, vata conditions (nervousness, anxiety, tremors, constipation, and light and interrupted sleep), fever, heart diseases, skin diseases, and neuroprotection. Sushruta (an ancient Indian physician, known as the main author of the treatise *The Compendium of Susruta*) used the plant in vomiting, indigestion, colic pain, and fever [6]. The roots and seeds are rich in pregnane glycosides of 3-Oxy sugars. The stem contains polyoxypregnane glycosides namely tenacigenoside I [10], tenacissoside C [11], tenacigenoside K, tenacigenosides G, tenacissoside H [11,12], marsdenoside B to marsdenoside H [13], and 11α-*O*-2-Methylbutyryl-12β-*O*-acetyltenacigenin B [14]. Its root is rich of polyoxypregnane glycosides, marstenacissides A1–A7, marstenacissides A8–A12 and marstenacissides B1–B9, marstenacissides B10–B17, and marsdenosides M [8,15]. The leaves are given for flatulence and as a remedy for gonorrhoea. The leaves and flowers are also used in cough, fever, and vomiting [16].

However, no studies on the toxicity of MT leaves have been described in the literature. Therefore, in the present investigation, we aimed to investigate the toxicity (both oral acute and subacute) of MT leaves in order to increase the confidence in their safety to humans to treat various ailments.

## 2. Materials and Methods

### 2.1. Collection and Identification of Plant Material

MT leaves were collected from the surrounding area of rural Moradabad during September 2016. The plant was identified and authenticated by Dr. Ashok Kumar, Botanist, School of Sciences, IFTM University, Moradabad, India, where the voucher specimen was deposited (03/MT/BOT) for future reference.

### 2.2. Sample Preparation

The plant material was collected in fresh condition. The plant material was air dried for two weeks. It was ground to a coarse powder with pestle and mortar. The coarse powder was further processed to fine powder with an electric blender.

#### Sample Extraction

Two hundred grams of the MT fine powdered sample were continuously extracted with 500 mL of 90% ethanol [7] for 48 h by using a Soxhlet apparatus (Modern Scientific Industries, Meerut, India) at 55°C. The extract was then evaporated under reduced pressure by using a rotary evaporator (Modern Scientific Industries) and further concentrated in a water bath at 55°C. The yield of the extract was 19.03% based on dry weight.

### 2.3. Experimental Animals

Male and female albino rats (*Rattus norvegicus*) weighing 130–160 g were used for the acute and subacute toxicology studies. The rats were obtained from the animal house, IFTM, Moradabad, India. The animals were acclimatized to laboratory conditions for sevendays prior to the experiments. The rats were maintained at a room temperature of 22–24 °C, with a 12 h light/dark cycle and humidity around (50 ± 5)%. During acclimatization, the rats were randomized into experimental and control groups and housed individually in sanitized polypropylene cages housed with sterile paddy husk as bedding. Animals were given free access to standard pellet diet and water ad libitum. All experimental procedures were in compliance with the Animal Ethical Committee, Committee for the Purpose of Control And Supervision of Experiments on Animals (CPCSEA) and were approved by University Ethical Committee with an approval number 2016/830/ac/Ph.D/08.

### 2.4. Acute Oral Toxicity Study

An acute oral toxicity study was performed according to the Organization of Economic Co-Operation and Development (OECD) guideline 420 for testing of chemicals [17]. Rats of both sexes (fasted for 16 h), aged 6–8 weeks old, were used. *Marsdenia tenacissima* extract (MTE) was dissolved in 10% Tween 20 and administered only once orally at a single dose of 5000 mg/kg at a rate of 20 mL/kg to both the sexes of rats (*n*= 12; six males, six females), whereas the control group only received 10% Tween 20 as a vehicle. All rats were then allowed free access to food and water and observed for 24 h, with special care given to first 4 h and once daily for 14 days for any signs of acute toxicity.

The visual observations of mortality, various changes in physical appearance, behaviour (salivation, lethargy), and any injury or illness were conducted once daily for 14 days. On the 15th day, all animals were anesthetized by intraperitoneal injection of ketamine. Blood samples were collected by cardiac puncture into EDTA containing tubes and non-heparinized tubes for haematological and biochemical analysis, respectively. Haematological and biochemical analyses were performed at Diagno Care Path Lab, Moradabad, Uttar Pradesh, India. Rats were then euthanized through intraperitoneal injection of ketamine. The organs, namely the liver, heart, spleen, lung, and kidney, were carefully excised and weighed. These organs were preserved in a fixation medium of 10% buffered formalin for histopathological study. The relative organ weight of each animal was calculated as follows:

Relative organ weight = (organ weight (g)/body weight of the animal on sacrifice day (g)) × 100

### 2.5. Subacute Toxicity Study

Subacute oral toxicity study was performed according to the Organization of Economic Co-Operation and Development (OECD) guideline 407 for testing of chemicals [18] and World Health Organization guideline [19]

Ten female rats were used to study the test. On the basis of results of acute toxicity study indicated that MTE extract was nontoxic at the dose level of 5000 mg/kg, subacute toxicity study of MTE at the doses of 250, 500, and 1000 mg/kg body weight were administered orally to fourgroups, respectively, at every 24 h for 28 days and controls received 10% Tween 20 as a vehicle at the same volume. Various toxic signs and observation, such as body weight, mortality, and food and water intake was monitored. After 28 days, all surviving animals were fasted overnight and anesthetized. The heparinized blood samples were collected for determining haematological parameters and theserum from non-heparinized blood was collected for determining clinical blood chemistry. Rats were then euthanized after blood collection and the internal organs (heart, liver, spleen, kidney, and lungs) were removed and weighed to determine the relative organ weights and observedfor any gross lesions. The internal organs were preserved in 10% buffered formaldehyde solution for histopathological study [20].

#### 2.5.1. Weekly Body Weight

The body weight of each rat was carefully monitored before study commencement, once weekly during the study, and on the day of sacrifice.

#### 2.5.2. Mortality and Toxic Signs

The visual observations of mortality, various changes in physical appearance, behaviour (sleepy, salivation, lethargy), and any injury or illness were conducted once daily for 28 days, especially after dosing and up to 4 h after dosing [21].

#### 2.5.3. Relative Organ Weight

On the 29th day, all the animalswere anesthetized by an intraperitoneal injection of ketamine. Blood samples were collected by cardiac puncture into EDTA containing tubes and non-heparinized tubes for haematological and biochemical analysis, respectively. Rats were then euthanized after blood collection and the internal organs (heart, liver, spleen, kidney, and lungs) were removed and weighed to calculate the relative organ weights (using the formula) and observed for any gross lesions. The internal organs were preserved in 10% buffered formaldehyde solution for histopathological study [20].

#### 2.5.4. Haematological Parameters

After collecting blood from cardiac puncture into EDTA containing tubes, various parameters were evaluated at Diagno Care Path Lab, Moradabad, Uttar Pradesh, India. The haematological parameters, like haemoglobin (Hb), red blood cell (RBC), PCV (packed cell volume), MCV (mean corpuscular volume), mean corpuscular haemoglobin (MCH), mean corpuscular haemoglobin concentration (MCHC), total white blood cells (WBCs), differential WBCs (neutrophil, lymphocyte, and monocyte), platelet count, red blood cell distribution unit (RDW), platelet distribution width (PDW), platelet large cell ratio (P-LCR), mean platelet volume (MPV), and procalcitonin (PCT) were estimated [22].

#### 2.5.5. Biochemical Estimations

Blood collected in non-heparinized tubes were then centrifuged at 3000 r/min for 10 min. The serum separated was analysed at Diagno Care Path Lab, Moradabad, Uttar Pradesh, India, for various parameters such as sodium, potassium, chloride, creatinine, urea, uric acid, total protein, albumin, globulin, albumin-globulin ratio, alkaline phosphatase (ALP), aspartate aminotransferase (AST), alanine aminotransferase(ALPT), and total bilirubin [20].

#### 2.5.6. Histopathology Study

The organs, namely liver, heart, spleen, lung, and kidney were carefully excised and weighed. These organs were preserved in a fixation medium of 10% buffered formalin for histopathological study. The organ paraffin sections were prepared, stained with haematoxylin and eosin, and processed for light microscope following the techniquein [23].

### 2.6. Statistical Analysis

All values were expressed as the mean ± SD (standard deviation) and the results were analysed statistically by one-way Analysis of Variance (ANOVA) followed by Tukey’s multiple comparison tests using statistical software- GraphPad Prism (GraphPad Software, Inc., La Jolla, CA, USA) version 5.0. *p* < 0.05 compared to control was considered to be statistically significant.

## 3. Results

### 3.1. Acute Oral Toxicity Study

**Figure 1:**
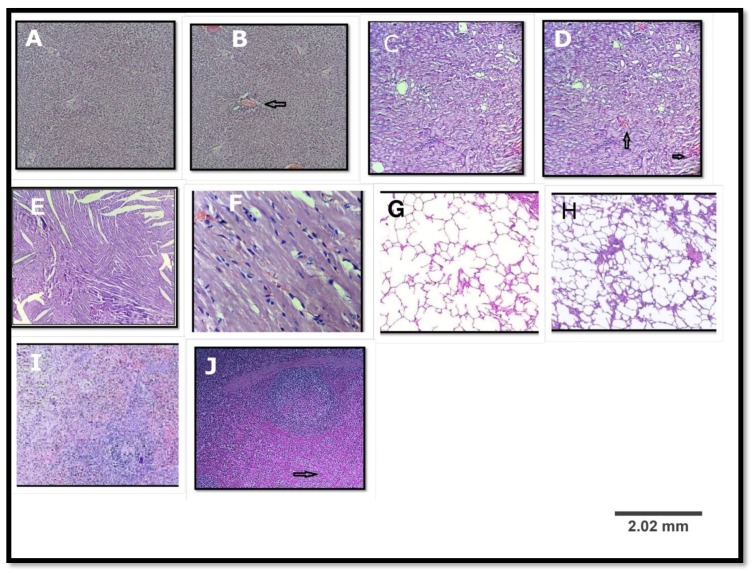
Histopathological examination of various organs of the rat in acute oral toxicity study; (**A**,**C**,**E**,**G**,**I**) are liver, kidney, heart, lung, and spleen of the control group; (**B**,**D**,**F**,**H**,**J**) are liver, kidney, heart, lung, and spleen of the treated group. (**B**) A hydrophobic degeneration along with mild congestion in portal vein (arrow); (**D**) mild congestion in intertubular blood vessels(arrow); (**F**) normal myocardium; (**H**) normal lung; and (**J**) mild congestion in red pulp (arrow). (Haematoxylin & Eosin, (H&E) ×100).

Ethanolic extract of *Marsdenia tenacissima* (MTE) at a dose of 5000 mg/kg produced no toxic effect on the behavioural responses of the treated rats (dosed once) and observed for 14 days. There were no signs of changes in the behaviour patterns, skin, eyes, salivation, and diarrhoea of the rats. Neither mortality nor significant weight loss was observed. There were generally no significant differences observed in the relative organ weight (Table 1). From the present study it was seen that there was no significant change in the haematological and biochemical parameters in the MTE treated group compared to control group (Table 2 and Table 3). The histopathological evaluations of various organs stained with haematoxylin and eosin revealed no significant differences (Figure 1). Although some differences has been observed, but the haematological and biochemical parameters showed no significant differences in the physiological parameters (Table 2 and Table 3). The differences appeared only in two treated rats. The rest of the rats (eight rats) showed no changes in their histopathology. The LD_50_ of this plant was, therefore, estimated to be more than 5000 mg/kg. 

### 3.2. Subacute Toxicity Test

#### 3.2.1. Weekly Body Weight

A weekly body weight was determined on initial (0) day, 9th, 18th, and 28th days of four groups. The first one is the control, Group I is MTE of 250 mg/kg, II MTE of 500 mg/kg, and the last group, named as Group III, is MTE of 1000 mg/kg. No significant changes in the body weight were observed (Table 4). 

#### 3.2.2. Clinical Observation and Mortality

Daily oral administration of MTE for 28 days did not produce any symptoms of toxicity in rats, including the highest dose tested at 1000 mg/kg body weight. No deaths or obvious clinical signs were found in any groups throughout the study. None of the rats showed signs of toxicity in their skin, fur, eyes, sleep, salivation, diarrhoea, and behaviour. The food and water consumptions of the treated rats, which were measured throughout the study, were not significantly different compared to controls.

#### 3.2.3. Relative Organ Weight

Relative organ weights of 28-day treated rats are shown in Table 5. The relative organ weight of each organ recorded at necropsy in the treatment groups did not show a significant difference (*p* > 0.05) compared to control (Table 5). 

#### 3.2.4. Haematological Parameters

The effects of subacute administration of MTE on haematological parameters are presented in Table 6. Most haematological parameters, likehaemoglobin, total RBCs, RDW, WBCs, neutrophils, lymphocytes, monocytes, andplatelet count, in treated rats were not significantly different from the control (Table 6). 

#### 3.2.5. Biochemical Analysis

The effects of subacute administration of MTE on biochemical parameters are presented in Table 7. The MTE had no effect on serum electrolytes (Na^+^, K^+^, and Cl^−^). The kidney function parameters, likeurea, creatinine, and uric acid, did not reveal any significant changes. No statistically significant differences in the liver function parameters like alanine aminotranferase (ALT), aspartate aminotransferase (AST), and alkaline phosphatase (ALP) were observed. Additionally, no relevant changes were found in total protein, albumin, and globulin (Table 7).

#### 3.2.6. Histopathological Study

**Figure 2:**
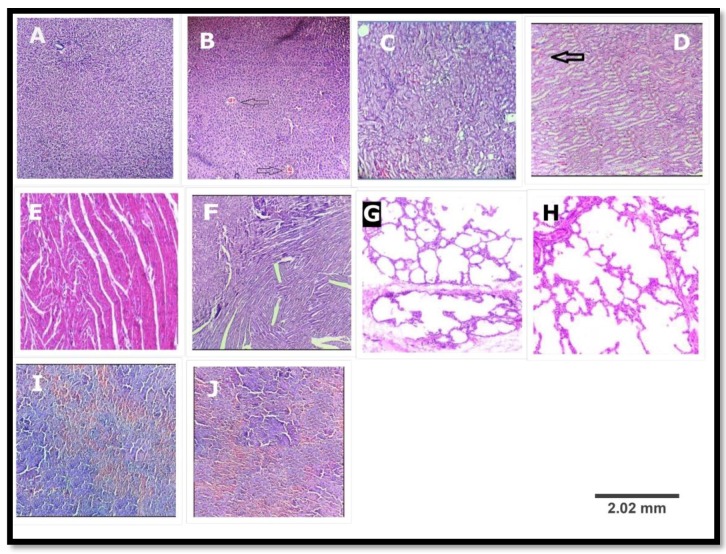
Histopathological examination of various organs of the rat in subacute oral toxicity study; (**A**,**C**,**E**,**G**,**I**) are liver, kidney, heart, lung and spleen of the control group; (**B**,**D**,**F**,**H**,**J**) are liver, kidney, heart, lung and spleen of treated group (MTE: 1000 mg/kg). (**B**) A hydropic degeneration along with mild congestion in portal vein (arrow); (**D**) mild congestion in intertubular blood vessels (arrow); (**F**) normal myocardium; (**H**) normal lung; and (**J**) mild congestion of red pulp. ((H,E) ×100).

Light microscopic examination of sections of various organs like liver, heart, spleen, lung, and kidney of control and treated groups showed a normal histology (Figure 2) and absence of any gross pathological lesions. Additionally, macroscopic examination of organs of treated rats revealed no abnormalities in the colour or texture when compared with the organs of the control group. Although some differences havebeen observed, the haematological and biochemical parameters showed no significant differences in the physiological parameters (Table 6 and Table 7). The differences appeared only in two treated rats. The rest of the rats (eight rats) showed no changes in their histopathology.

## 4. Discussion

For centuries, herbal medicines and their formulations have been considered to be safe and effective due to their negligible side effects. This assumption may have influenced the indiscriminate use of these formulations to a large extent amongst the rural populace. These formulations are usually administered over a long period of time without proper dosage monitoring by the experts and lack of awareness of the toxic effects that might result from such prolonged usage [24]. Therefore, scientific knowledge towards oral toxicity is much needed, which will not only help identify doses that could be used subsequently, but also to reveal the possible clinical signs elicited by agents under investigation. Regardless of the pharmacological benefits of the *Marsdenia tenacissima*, detailed knowledge about subacute toxicity of this medicinal plant is lacking. Hence, the current study was undertaken to evaluate and focus on the acute and subacute toxicity of *Marsdenia tenacissima* leaves in an animal model.

In screening natural products for pharmacological activity, the evaluation of the toxic characteristics of medicinal products (extract, isolated compounds, and formulation) is usually a preliminary step. During such evaluation, the determination of LD_50_ is usually an initial step to be conducted. The acute toxicity study may provide initial information on the mode of toxic action of an agent, acts as the basis for classification and labelling, and helps in deciding the dose of novel compounds in animal studies. Moreover, if a high dose (e.g., 5000 mg/kg) is found to be survivable, no further acute testing will be conducted [25]. In this study, MTE leaves at a dose of 5000 mg/kg had no adverse effect on the treated rats in up to 14 days of observation. There were no significant changes in the weight and the organs of the rats. The hematological parameters between control and treated groups showed the extract was non-toxic to the haemopoietic system. Additionally, most of the biochemical parameters were not altered. No relevant changes were found in levels of ALT, AST, ALP, or creatinine, which are good indicators of liver and kidney functions. No gross lesions were found in histopathology examinations. Therefore, this study indicates that the leaves of MTE do not cause acute toxicity effects at the dose tested and with LD_50_ values greater than 5000 mg/kg. According to the OECD, under chemical labelling and classification MTE may be assigned a class 5 status (LD_50_ > 5000 mg/kg), which was the lowest toxicity class. Since no toxic effects were found during the acute toxicity study, further study was conducted to evaluate the subacute toxicity of MTE leaves up to 28 days to prepare inclusive toxicological records on this plant.

Subacute studies provide information on dosage regimens, target organ toxicity, and identify observable adverse effect that may affect the average life span of experimental animals. Consequently, in this study, the leaves of MTE wereevaluated in rats at doses of 250, 500, and 1000 mg/kg for 28 days. The body weight changes serve as a sensitive indication of general health status of animals [26]. After 28 days of treatment of the extract, all the animals exhibited a normal increment in body weight. It can be stated that leaves of MTE did not interfere with the normal metabolism of animals. The significant increment in food and water intake is considered as being responsible for augmentation in body weight gain. 

Similarly, no significant changes in the weight of the heart, liver, lung, spleen and kidney were observed, suggesting that administration of MTE leaves at subacute oral doses produces no effect on the normal growth. The protocol of weighing relative organs in toxicity studies includes their sensitivity to predict toxicity and it correlates well with histopathological changes [27]. The results of this study revealed no significant changes in the relative organ weight of control and treated groups which showed that none of the organs were adversely affected, nor showed any signs of toxicity throughout the study.

The haematological parameters can be used to determine the blood relating functions of plant extract. The haemopoietic system is one of the most sensitive targets of toxic compounds and an important index of physiological and pathological status in both humans and animals. The extract indicated a non-significant difference on the RBC indices which suggested that the MTE does not affect erythropoiesis, morphology, or osmotic fragility of red blood cells [28]. WBC’s are the first line of cellular defines that respond to infectious agents, tissue injury, or any inflammation. Furthermore, no significant changes were observed in neutrophils, lymphocytes, and monocytes in the leaves of MTE suggesting that the extract might not have exerted challenge on the immune system of the animals.

Evaluation of serum biochemistry was done to identify the possible alterations in renal and hepatic functions affected by extract. Total protein, albumin, globulin, and total bilirubin also affecting the hepatocelluar and secretory functions of the liver. The lack of significant alterations in the levels of ALT, AST, ALP, creatinine, and uric acid, which are good indicators of liver and kidney functions [29], suggests that sub-chronic administration of extract neither altered hepatocytes and kidneys of rats, nor the normal metabolism of the animals. These observations were further confirmed by the histological assessment of the organs showed in Figure 2. Based on the results found in our study, we concluded that leaves of *Marsdenia tenacissima* ethanolic extract was safer and non-toxic and could be well used for pharmacological and therapeutic purposes. Moreover, no work has been reported on its leaves (i.e., isolation of chemical constituents and their characterization). Very few species of *Marsdenia* possess neurological toxicity. These species are *Marsdenia hilariana* and *Marsdenia megalantha* [30].

## 5. Conclusions

In the light of these findings, we may conclude that *Marsdenia tenacissima* leaf extract is not toxic in all doses studied herein and did not produce any evident symptoms in the acute and subacute oral toxicity studies. The histology examination revealed no remarkable changes in the internal organs, like kidney, liver, spleen, lung, and heart of the rats, in both control and treated groups. Furthermore, the data of acute and subacute toxicity studies on this plant were obtained in order to increase the confidence in its safety to humans for the use in the development of pharmaceuticals. The studies also need further experimental activities, like subchronic toxicity study, the effect of the extract on pregnant rats, foetuses, and their reproductive capacity to complete the safety profile of this plant.

## Figures and Tables

**Table 1:** The relative organ weight of rats treated with a single dose of *Marsdenia tenacissima* extract (MTE) for 14 days.

Organs	Control	MTE (5000 mg/kg)
Heart	0.46 ± 0.02	0.43 ± 0.01^ns^
Liver	3.32 ± 0.13	3.28 ± 0.11 ^ns^
Spleen	0.31 ± 0.02	0.33 ± 0.02 ^ns^
Kidney	0.84 ± 0.03	0.86 ± 0.01 ^ns^
Lungs	0.79 ± 0.05	0.77 ± 0.02 ^ns^

Values are expressed as the mean ± SD (standard deviation) (*n* = 12; for each group, six male, six female); One-way ANOVA followed by Tukey’s multiple comparison test. *p* > 0.05. ^ns^ Not significant. Relative organ weight was calculated as (organ weight (g)/body weight of animal on sacrifice day (g)) × 100.

**Table 2:** Effect of MTEon haematological parameters in acute oral toxicity study.

Parameter	Unit	Control	MTE (5000 mg/kg)
Haemoglobin (Hb)	g/L	140.23 ± 3.11	140.77 ± 3.02 ^ns^
Total red blood cells (RBC’s)	10^12^/L	7.52 ± 0.23	7.31 ± 0.31 ^ns^
Packed cell volume (PCV)	L/L	0.43 ± 0.01	0.46 ± 0.00 ^ns^
Mean corpuscular volume (MCV)	fL	54.3 ± 0.00	54.21 ± 0.77 ^ns^
Mean corpuscular haemoglobin (MCH)	pg	18.55 ± 0.66	18.01 ± 0.42 ^ns^
Mean corpuscular haemoglobin concentration (MCHC)	g/L	328 ± 4.72	327.03 ± 4.13 ^ns^
Total white blood cells (WBC’s)	10^9^/L	7.52 ± 0.22	9.00 ± 1.3 ^ns^
Neutrophils	%	12.13 ± 1.03	12.07 ± 1.00 ^ns^
Lymphocytes	%	85.41 ± 1.92	82.02 ± 2.7 ^ns^
Monocytes	%	2.38 ± 0.00	2.31 ± 0.07 ^ns^
Eosinophils	%	1.01± 0.01	1.09 ± 0.02 ^ns^
Platelet count	10^9^/L	870 ± 10.71	865.00 ± 17.11 ^ns^

Values are expressed as the mean ± SD (*n* = 12; for each group, six male, sixfemale). One-way ANOVA followed by Turkey’s multiple comparison test. *p* > 0.05. ^ns^ Not significant.

**Table 3:** Effect of MTE on biochemical parameters in acute oral toxicity study.

Parameter	Unit	Control	MTE (5000 mg/kg)
Sodium	mmol/L	138.00± 0.20	138.20 ± 0.08 ^ns^
Potassium	mmol/L	6.12 ± 0.32	6.33 ± 0.35 ^ns^
Chloride	mmol/L	104.00 ± 0.27	104.30 ± 0.01 ^ns^
Urea	mmol/L	6.57 ± 0.34	6.36 ± 0.20 ^ns^
Creatinine	µmol/L	45.07 ± 0.33	45.12± 0.48 ^ns^
Uric acid	mmol/L	0.17 ± 0.03	0.19 ± 0.01 ^ns^
Total protein	g/L	69.37 ± 0.21	69.53 ± 0.43 ^ns^
Albumin	g/L	36.73 ± 0.67	37.13 ± 0.71 ^ns^
Globulin	g/L	32.64 ± 0.47	32.40 ± 0.51 ^ns^
Albumin/globulin ratio		1.12 ± 0.04	1.09 ± 0.05 ^ns^
Alkaline phosphatase (ALP)	U/L	135 ± 8.32	135.04 ± 9.71 ^ns^
Aspartate aminotransferase (AST)	U/L	74.31 ± 4.31	74.03 ± 3.39 ^ns^
Alanine aminotranferase (ALT)	U/L	45.41 ±0.71	45.81 ± 0.52 ^ns^

Values are expressed as the mean ± SD (*n* = 12; for each group, six male, six female). One-way ANOVA followed by Tukey’s multiple comparison test. *p* > 0.05. ^ns^ Not significant.

**Table 4:** The effect of MTE leaves on body weight of rats (g) at different days.

Group	Doses	Weight			
		Initial day	9th day	18th day	28th day
Control	-	134.21 ± 4.01	139.54 ± 5.24	145.56 ± 4.78	147.21 ± 4.85
I	250 mg/kg	138.23 ± 5.22	145.36 ± 5.78	149.25 ± 6.21	153.54 ± 5.74
II	500 mg/kg	142.22 ± 6.24	148.26 ± 5.01	152.21 ± 5.89	155.87 ± 5.81
III	1000 mg/kg	140.25 ± 3.22	146.28 ± 3.89	150.66 ± 4.86	153.24 ± 4.01

Group I-III: ethanolic extract with different doses; one-way ANOVA followed by Tukey’s multiple comparison test. *p* > 0.05 (not significant).

**Table 5:** The relative organ weight of rats treated with different doses of MTE for 28 days.

Organs	Control	MTE (250 mg/kg)	MTE (500 mg/kg)	MTE (1000 mg/kg)
Heart	0.45 ± 0.05	0.44 ± 0.01^ns^	0.42 ± 0.01 ^ns^	0.43 ± 0.02 ^ns^
Liver	3.03 ± 0.10	3.24 ± 0.15 ^ns^	3.14 ± 0.04 ^ns^	3.15 ± 0.08 ^ns^
Spleen	0.33 ± 0.02	0.30 ± 0.01 ^ns^	0.29 ± 0.02 ^ns^	0.28 ± 0.01 ^ns^
Kidney	0.74 ± 0.02	0.77 ± 0.01 ^ns^	0.73 ± 0.01 ^ns^	0.74 ± 0.03 ^ns^
Lungs	0.85 ± 0.06	0.91 ± 0.09 ^ns^	0.86 ± 0.02 ^ns^	0.88 ± 0.01 ^ns^

Values are expressed as the mean ± SD (*n* = 10; female rats); Relative organ weight was calculated as (organ weight/body weight) × 100; *p* > 0.05 using one-way ANOVA followed by Tukey’s multiple comparison test; ^ns^ Not significant.

**Table 6:** Effect of MTE on haematological parameters in the subacute oral toxicity study.

Parameter	Unit	Control	250 mg/kg	500 mg/kg	1000 mg/kg
Haemoglobin (Hb.)	g/L	150.33 ± 0.71	146.11 ± 0.11	147.31 ± 0.27	149.52 ± 0.51
Total red blood cells (RBC’s)	10^12^/L	7.52 ± 0.18	7.62 ± 0.31 ^ns^	7.20 ± 0.26 ^ns^	7.49 ± 0.22 ^ns^
Packed cell volume (PCV)	L/L	0.46 ± 0.01	0.43 ± 0.07 ^ns^	0.43 ± 0.02 ^ns^	0.42± 0.01 ^ns^
Mean corpuscular volume (MCV)	fL	55.41 ± 0.77	54.33 ± 0.21 ^ns^	54.81 ± 0.11 ^ns^	55.01 ± 0.03 ^ns^
Mean corpuscular haemoglobin (MCH)	pg	17.67 ± 0.17	17.20 ± 0.22 ^ns^	17.44 ± 0.14 ^ns^	17.57 ± 0.24 ^ns^
Mean corpuscular haemoglobin concentration (MCHC)	g/L	310.22 ± 0.04	313.07 ± 0.14 ^ns^	314.11 ± 0.23 ^ns^	311.71 ± 0.44 ^ns^
Total white blood cells (WBC’s)	10^9^/L	7.23 ± 0.17	7.01 ± 0.11 ^ns^	7.45 ± 0.19 ^ns^	7.66 ± 0.29 ^ns^
Neutrophils	%	12.71 ± 1.01	12.70 ± 1.13 ^ns^	12.60 ± 1.11 ^ns^	12.03 ± 1.79 ^ns^
Lymphocytes	%	86.11 ± 2.91	86.00 ± 2.01 ^ns^	86.51 ± 1.13 ^ns^	86.33 ± 1.00 ^ns^
Monocytes	%	2.10 ± 0.04	2.91 ± 0.11 ^ns^	2.61 ± 0.65 ^ns^	2.17 ± 0.31 ^ns^
Platelet count	10^9^/L	833.60 ± 48.20	838.51± 42.31 ^ns^	841.11 ± 41.11 ^ns^	840.08 ± 53.33 ^ns^
Red blood cell distribution unit (RDW)	%	14.37 ± 1.12	13.11 ± 1.93 ^ns^	13.49 ± 1.10 ^ns^	13.33 ± 1.09 ^ns^
Platelet distribution width (PDW)	fL	11.5 ± 0.01	10.91 ± 0.01 ^ns^	10.59 ± 0.04 ^ns^	9.76 ± 0.07 ^ns^
Platelet large cell ratio (P-LCR)	%	14.51 ± 0.11	14.70 ± 0.22 ^ns^	13.01 ± 0.22 ^ns^	13.71 ± 0.23 ^ns^
Mean platelet volume (MPV)	fL	7.72 ± 0.04	7.64 ± 0.08 ^ns^	7.27±0.01 ^ns^	7.57 ± 0.01 ^ns^
Procalcitonin (PCT)	%	0.99 ± 0.23	0.75 ± 0.37 ^ns^	0.68 ± 0.41 ^ns^	0.64 ± 0.44 ^ns^

Values are expressed as the mean ± SD (*n* = 10; for each group); *p* > 0.05 using one-way ANOVA followed by Tukey’s multiple comparison test; ^ns^ Not significant.

**Table 7:** Effect of MTE on biochemical parameters in the subacute oral toxicity study.

Parameter	Unit	Control	250 mg/kg	500 mg/kg	1000 mg/kg
Sodium	mmol/L	137.00 ± 0.44	135.20 ± 0.94 ^ns^	133.70 ± 0.86 ^ns^	134.30 ± 0.56 ^ns^
Potassium	mmol/L	6.37 ± 0.01	6.83 ± 0.09 ^ns^	6.39 ± 0.04 ^ns^	6.77 ± 0.13 ^ns^
Chloride	mmol/L	102.83 ± 0.03	102.70 ± 0.05 ^ns^	102.11 ± 0.03 ^ns^	101.00 ± 0.01 ^ns^
Urea	mmol/L	6.65 ± 0.21	6.12 ± 0.17 ^ns^	6.98 ± 0.18 ^ns^	6.81 ± 0.10 ^ns^
Creatinine	µmol/L	47.30 ± 0.52	44.17 ± 0.69 ^ns^	46.1 ± 0.61 ^ns^	46.79 ± 0.49 ^ns^
Uric acid	mmol/L	0.16 ± 0.04	0.15 ± 0.01 ^ns^	0.14 ± 0.09 ^ns^	0.15 ± 0.02 ^ns^
Total protein	g/L	68.50 ± 0.90	68.30 ± 0.86 ^ns^	69.00 ± 1.14 ^ns^	68.81 ± 0.90 ^ns^
Albumin	g/L	36.67 ± 0.03	37.21 ± 0.03 ^ns^	38.77 ± 0.01 ^ns^	38.92 ± 0.03 ^ns^
Globulin	g/L	31.83 ± 0.05	31.09 ± 0.09 ^ns^	30.23 ± 0.09 ^ns^	29.89 ± 0.07 ^ns^
Albumin/globulin ratio		1.15 ± 0.00	1.19 ± 0.01 ^ns^	1.28 ± 0.01 ^ns^	1.30 ± 0.01 ^ns^
Alkaline phosphatase (ALP)	U/L	132.83 ± 6.17	133.70 ± 10.12 ^ns^	134.65 ± 12.92 ^ns^	135.00 ± 11.33 ^ns^
AST	U/L	71.00 ± 6.91	74.00 ± 6.02 ^ns^	75.21 ± 7.29 ^ns^	77.00 ± 6.24 ^ns^
ALT	U/L	48.67 ± 1.17	44.21 ± 2.42 ^ns^	48.21 ± 2.71 ^ns^	50.00 ± 1.23 ^ns^
Bilirubin (Total)	mg/dL	0.35 ± 0.01	0.31± 0.00 ^ns^	0.34 ± 0.01 ^ns^	0.45 ± 0.02 ^ns^

Values are expressed as the mean ± SD (*n* = 10; for each group); *p* > 0.05 using one-way ANOVA followed by Tukey’s multiple comparison test; ^ns^ Not significant.
